# Bacterial transcription during growth arrest

**DOI:** 10.1080/21541264.2021.1968761

**Published:** 2021-09-06

**Authors:** Megan Bergkessel

**Affiliations:** School of Life Sciences, University of Dundee, Dundee, UK

## Abstract

Bacteria in most natural environments spend substantial periods of time limited for essential nutrients and not actively dividing. While transcriptional activity under these conditions is substantially reduced compared to that occurring during active growth, observations from diverse organisms and experimental approaches have shown that new transcription still occurs and is important for survival. Much of our understanding of transcription regulation has come from measuring transcripts in exponentially growing cells, or from *in vitro* experiments focused on transcription from highly active promoters by the housekeeping RNA polymerase holoenzyme. The fact that transcription during growth arrest occurs at low levels and is highly heterogeneous has posed challenges for its study. However, new methods of measuring low levels of gene expression activity, even in single cells, offer exciting opportunities for directly investigating transcriptional activity and its regulation during growth arrest. Furthermore, much of the rich structural and biochemical data from decades of work on the bacterial transcriptional machinery is also relevant to growth arrest. In this review, the physiological changes likely affecting transcription during growth arrest are first considered. Next, possible adaptations to help facilitate ongoing transcription during growth arrest are discussed. Finally, new insights from several recently published datasets investigating mRNA transcripts in single bacterial cells at various growth phases will be explored. Keywords: Growth arrest, stationary phase, RNA polymerase, nucleoid condensation, population heterogeneity

## Introduction

Francois Jacob, Andre Lwoff, and Jacques Monod received the Nobel prize in physiology or medicine in 1965 for their pioneering studies on the regulation of the *lac* operon of *Escherichia coli* [1,2]. This work was foundational for the study of transcription regulation throughout all kingdoms of life. However, prior to working on the molecular genetics of the *lac* operon, Monod had been focused on a quantitative framework for the study of bacterial growth. He made the observation that under some conditions the growth rate of a bacterial culture could be related to the concentration of a limiting nutrient using the same mathematical equations used to describe rates of enzyme catalysis as a function of substrate concentration by Leonor Michaelis and Maud Menten [3,4]. The required conditions are that growth is balanced (each new cell had the same composition as its parent cell) and steady state (a non-equilibrium condition in which there is flux through metabolic pathways, but concentrations of reactants and products remain constant through continual appearance of new reactants and removal of new products). These conditions are satisfied, at least to a first approximation from the perspective of the cell, during exponential growth.

Exponential growth has remained the “standard state” in which to study mechanisms of transcriptional regulation since those early days, and this approach continues to be useful. For example, an elegant systems-level understanding of how transcriptional regulatory mechanisms contribute to optimal “proteome allocation” during exponential growth at different rates has recently emerged [5,6]. A focus on studying the transcriptional machinery during exponential growth at fast rates has also revealed exquisite choreography that allows the RNA polymerase (RNAP) and DNA polymerase to share the same DNA substrate and avoid conflict, even while simultaneously producing 60,000 new ribosomes and a new chromosome within a 20-minute doubling time [7]. However, the next-generation sequencing revolution has helped to remind us that bacteria are stuffed into virtually every crevice of every organism and object on our planet. Most of these estimated 10^30^ creatures [8] must not actually spend much time in balanced, steady-state, exponential growth, but instead grow slowly and divide rarely. To fully understand the bacterial transcriptional machinery, deeper insight into its functioning in these ubiquitous growth-arrested states is required.

**Figure 1.**
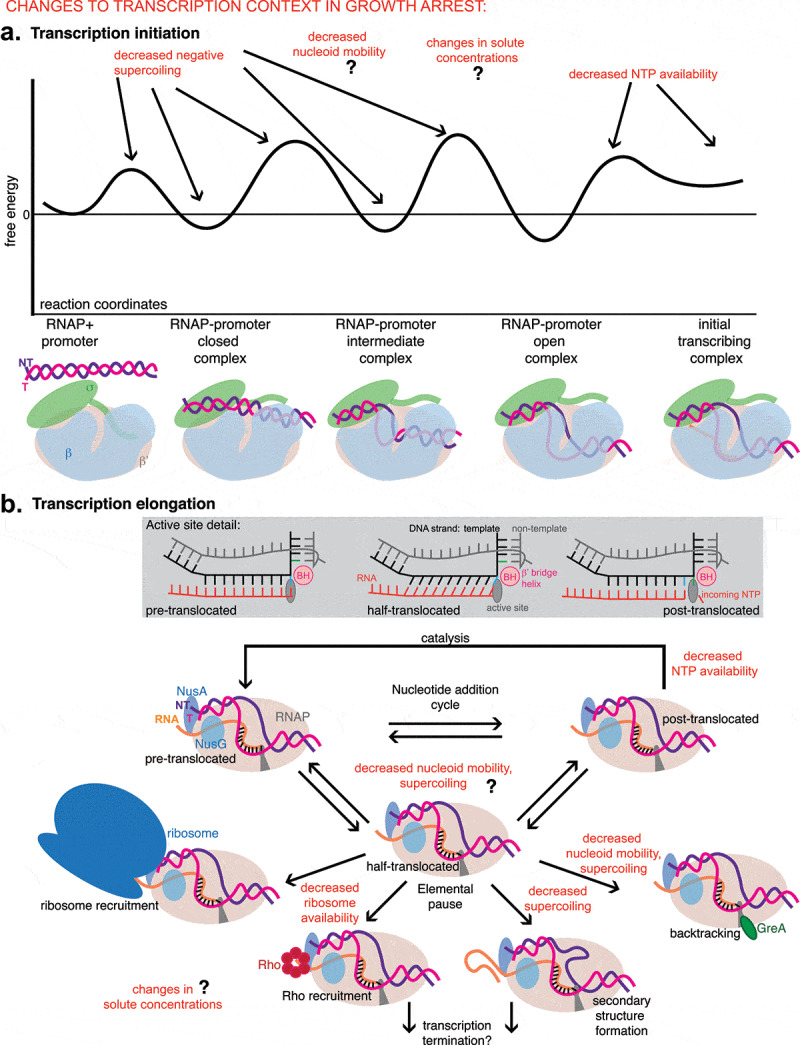
Transitions involved in transcription initiation and elongation, and potential impacts of growth arrest on them. A. The process of transcription initiation can be described as an energy landscape in which several transitions present barriers to progression toward entry into the elongation phase of transcription. Changes to the chromosome and nucleoid environment during growth arrest (indicated in red, potential positive or negative impacts depicted by arrows) also alter the energy landscape in a promoter-dependent manner, potentially raising or lowering local maxima and/or minima. Cartoons below depict stable intermediates (local energy minima) that have been observed in cryoEM or crystal structures. Other changes in nucleoid mobility and solute concentrations might affect many steps in initiation, but further investigations are needed. This figure is based on ideas presented in multiple works [13–15] B. Transcription elongation consists of a repeating nucleotide addition cycle. Detailed schematics of the active site conformations are shown in the gray box. Blue and green colored bases in the DNA template strand indicate the shifting register. Between the pre- and post- translocated states, the elongation complex can enter an off-pathway, half-translocated “elemental pause” state, from which diverse interactions can affect the fate of the elongation complex. Changes during growth arrest (indicated in red) can in theory impact the likelihood of these interactions. For much more detail, see recent excellent reviews [10,11]

Despite the traditional sense that exponential growth is the “standard state” for studying transcription, much of the experimental methodology that has been employed has no requisite ties at all to steady state growth. Reconstituted *in vitro* transcription reactions are often carried out under conditions that differ from what would occur in a rapidly growing cell for practical reasons, and a purified and crystallized or vitrified protein used in structural studies has lost all connection to cellular metabolism and growth. Therefore, many of the conclusions drawn from these *in vitro* mechanistic studies should in theory be equally applicable to consideration of non-growing states as they are to consideration of rapid growth. For mechanisms of transcription initiation, elongation, and pausing, elegant formalisms have emerged that should serve as useful tools for considering these processes under any growth condition (Figure 1). These topics have been recently and beautifully reviewed elsewhere [9–11], but a brief mention here of some key concepts will be helpful.

Transcription initiation in bacteria occurs when the RNA polymerase holoenzyme, consisting of core (α_2_ββ’ω) and sigma (σ) subunits, is recruited to a promoter sequence via specific contacts of the sigma factor and DNA. Between initially contacting the DNA and rapidly adding nucleotides to an elongating RNA transcript, the RNA polymerase must bend and unwind approximately 12 bases of the DNA duplex to form the transcription bubble and situate the template strand in the active site of the enzyme, deep in the main channel between the β and β’ subunits. This process requires the RNAP/promoter complex to pass through several intermediate conformations, which can be described by an energy landscape that is unique to the combination of sequence elements and regulatory factors present at the initiation event [12,13]. The energy landscape framework for considering transcription initiation has recently been refined by new structural, biochemical, and theoretical work from multiple organisms and is useful for considering the impact of changes in conditions and regulatory context on transcription initiation rates, including during growth arrest [14,15] (Figure 1a).

Once a few nucleotides have been added to the nascent transcript, the sigma factor can dissociate from the core RNAP and transcription elongation factors (NusA and NusG) usually join [16]. Over the past decade it has become increasingly clear that although RNAP is a highly processive enzyme, able to add thousands of nucleotides to a growing RNA in bacteria without dissociating from its template, it does not proceed at a constant rate. Instead, it pauses frequently, for highly variable amounts of time, and in a range of different conformations [17–19]. Although a transcription elongation complex in isolation is an extremely stable entity [20], during transcription *in vivo* its entry into an off-pathway “elemental” paused state, in which the nascent transcript is translocated relative to the active site but the template DNA is not, is highly sensitive to sequence context [19]. From this paused state, interactions of the nucleic acids with ribosomes [17], regulators like Rho [21–23], or themselves (e.g. formation of hairpins in the nascent RNA [24] or even in the non-template DNA strand [25]) can strongly influence the fate of the elongation complex (Figure 1b).

The goal of this review is to explore how this current understanding of transcriptional processes can be applied to the cellular context of the protracted growth arrests that are ubiquitous in the microbial world. First, the impacts on transcription of changes to cellular physiology that occur during growth arrest are discussed, and then possible adaptations to these changes are described. Finally, new methods to measure low levels of transcription in single cells will be briefly discussed, and preliminary conclusions from such measurements examined. Growth-arrested populations of bacteria show very low average levels of activity and high heterogeneity, even among well-mixed and genetically identical populations, so sensitive single-cell methods will be key to understanding their physiology.

## The transcriptional challenges of growth arrest

Bacteria cease growing for a wide variety of reasons, which usually ultimately cause energy limitation and/or a lack of building blocks for biosynthesis [26]. Historically, the most common models for studying growth arrest have been *E. coli* cells grown into stationary phase in rich media such as Lysogeny Broth (LB) (where carbon is likely limiting [27]), or starved for carbon for variable periods of time in a more defined media. Unless otherwise indicated, examples cited throughout this review are from such models. Perhaps the most obvious change occurring in these growth-arrested states is a decrease in cell size that has been attributed to “reductive divisions” taking place during the transition from growth to growth arrest [27]. This process is a clear deviation from the principle of balanced growth, a consequence of the need to complete open rounds of DNA replication and subsequent cell division while biosynthetic rates for ribosomes and other cellular components are downregulated [28,29]. The resulting changes in gross morphology have the potential to impact transcription during the growth arrest that follows the completion of the reductive divisions, because the DNA becomes denser and more compacted even though the nucleoid occupies a slightly greater fraction of the cellular volume as growth rates slow toward growth arrest [30,31]. Recent studies have suggested that nutrient downshift can result in a modest decrease in cytoplasmic volume even in the absence of reductive divisions, leading to a further increase in cellular density [32]. Similar phenomena have also been observed in eukaryotic microbes such as yeast and have been proposed in both systems to contribute to increased macromolecular crowding, decreased mobility, and increased rigidity of both the chromosome(s) and the cytoplasm [33,34]. This decreased mobility can directly impact diffusion within the nucleoid, affecting the ability of transcription factors to diffuse away from their encoding loci [35], for example, but it does not completely exclude RNAP or ribosomes from the nucleoid region [36]. Transcription and translation do still occur at much reduced rates, but the motions of RNAP and ribosomes are in a different physical environment and may be subject to higher physical resistance.

In addition to changes in macromolecular crowding, the topological characteristics of the DNA change during growth arrest. The details of bacterial chromosome topology are outside the scope of this review but have been reviewed previously [37–41]. On average, the DNA of a bacterial chromosome (or plasmid) is underwound relative to the natural twist of the DNA double helix (10.4–10.5 bases per full turn), a condition referred to as negative superhelicity or supercoiling. When the DNA double helix is locally unwound to open a transcription or replication bubble and allow a polymerase to translocate, the helix becomes underwound (negatively supercoiled) upstream and overwound (positively supercoiled) downstream of the polymerase [42]. Negative supercoils are re-introduced downstream of the polymerase in an ATP-dependent reaction that requires transiently breaking both DNA strands by DNA gyrase [43], while topoisomerase I relaxes negative supercoils behind polymerases with no ATP requirement. Average superhelical density has been shown to depend on rates of gyrase activity and transcription elongation and tends to be highest in rapidly growing cells [44].

The average superhelical density *in vivo* in different growth conditions has been estimated by extraction and gel analysis of plasmids [39]. However, when considering effects on transcription, the local topological characteristics of the transcribed region are relevant, and much more difficult to measure [44]. Supercoils can diffuse along the length of the DNA until impeded by a topological barrier. Many protein–DNA interactions (including an RNAP elongation complex) can act as topological barriers to diffusion of superhelical density [45]. In fact, the lower-mobility and higher-density state of the nucleoid during growth arrest may also affect the distribution of superhelical density by impeding rotation of the DNA and bound proteins. A new method to detect positively supercoiled regions of the chromosome *in vivo* may be useful for investigating these questions [46].

Local superhelical density can strongly impact transcriptional activity in several ways. First, the energetic barrier to melting the DNA helix during open complex formation can be decreased by negative superhelicity or increased by positive superhelicity [47]. Additionally, this under- or overtwisting of the DNA helix can affect the alignment of the −10 and −35 sites that are recognized by the sigma factor [48]. Changes in superhelicity can affect binding of transcription factors and nucleoid associated proteins, and vice versa [49], and these impacts are all sensitive to specific sequence context. For example, decreased negative supercoiling induces the DNA gyrase promoter but represses the topoisomerase I promoter, thus contributing to topological homeostasis in multiple organisms [48,50]. Promoters that are highly active during rapid growth (such as the ribosomal RNA promoters) are repressed by a decrease in negative supercoiling in both *E. coli* [51] and *Bacillus subtilis* [52]. Trapping of positive supercoils in front of RNAP can also impact transcription elongation, by favoring pausing and backtracking [53]. Finally, supercoiling can facilitate interactions among transcribing polymerases across multiple kb of DNA in growing cells [54]; whether the potential for these types of interactions is lost in growth arrested cells where many fewer polymerases are transcribing at the same time remains to be investigated. In general, maintenance of the negative superhelicity of the chromosome is energy-requiring and lowers the barrier to transcription initiation. During growth arrest, lower energy availability and transcriptional activity contribute to a topological state of the DNA that is less conducive to transcriptional activity in a self-reinforcing cycle.

In addition to biophysical changes to large macromolecules during growth arrest, changes to concentrations of small molecules could also affect transcriptional activity. Potassium and glutamate, the most abundant ionic species in the bacterial cytoplasm, are known to directly impact DNA–protein interactions and protein–protein interactions among proteins of the transcriptional machinery [55]. Intracellular potassium levels are actively modulated by regulatory mechanisms that are sensitive to the metabolic state of the cell. In many Gram-positive bacteria, this regulation involves the small signaling molecule cyclic di-AMP [56], while in *E. coli* the nitrogen-sensing branch of the phosphotransferase signal transduction system (PTS^Ntr^), which transfers phosphate from phosphoenolpyruvate to the effector protein PtsN in a multistep relay, plays an important role [57,58]. Increased intracellular potassium was shown to increase σ^38^ (a.k.a. RpoS, the general stress sigma factor) association with RNAP and expression from σ^38^ -dependent promoters, while decreasing σ^70^ (RpoD, the housekeeping sigma factor) association and σ^70^ -dependent transcription [58]. Polyamines appear to be imported and/or synthesized during growth arrest and biofilm formation [59] and can directly impact nucleoid compaction and gyrase activity [60,61].

The nucleotide substrates of RNAP are also affected by growth arrest, by complex regulatory mechanisms. It is non-trivial to measure intracellular concentrations of NTPs [62], but most measurements suggest that changes to NTP concentrations are relatively modest, decreasing no more than 2-4-fold during stationary phase or an induced stringent response [63–66]. Interestingly, during prolonged starvation of a marine *Vibrio sp*., the intracellular ATP concentration was reported to exceed the starting level after about 2 weeks [67], perhaps an impressive display of the robustness of homeostasis-preserving mechanisms. Numerous examples of sensitivities of transcriptional processes to nucleotide concentrations have been described, but it remains unclear how much they contribute to regulation during protracted growth arrest. The best-studied example is probably the regulation of transcription initiation at the rRNA (*rrn*) P1 promoters in *E. coli*. Especially in the presence of the small-molecule alarmone (p)ppGpp and global regulator DksA, the open complex formed at these promoters is exceptionally unstable, and sensitive to the concentration of the initiating nucleotide (ATP) over a physiologically relevant range [68,69]. However, downregulation of these promoters is most relevant at the transition to stationary phase, after which the *rrn* P2 promoters (which are less sensitive to NTP concentration) are more likely to drive the low levels of necessary transcription [64]. In *B. subtilis*, initiation at the rRNA promoters also appears to be sensitive to the concentration of the initiating nucleotide despite the fact that (p)ppGpp does not bind RNAP, but as in *E. coli*, different rRNA promoters show different degrees of sensitivity [70,71].

Escapes from transcription elongation pauses in some contexts also appear to be sensitive to NTP concentration [19,72,73]. However, this may not be due to a direct effect of NTP concentration on the paused state itself. The elemental pause that is the proposed intermediate for many regulatory decisions during transcription elongation does not appear to be compatible with nucleotide addition, and NTP concentration does not have any impact on escape from that state [19]. Instead, decreased nucleotide concentrations might increase the amount of time the polymerase spends in the post-translocated state before nucleotide addition, which would in theory increase the probability of sampling the half-translocated elemental pause state (Figure 1b). Even this effect may only rarely be relevant *in vivo*, as the *K_m_* values for addition of nucleotides other than UTP during elongation have been measured to be much lower than the concentrations observed in cells [73].

Although flux through RNA biosynthetic pathways (transcription) decreases by at least an order of magnitude during growth arrest, cellular regulatory mechanisms maintain NTP pools within a narrower range [65,66,74,75]. Other mechanisms, such as the changes to the nucleoid composition and DNA topology discussed above, contribute substantially to holding transcription levels low without completely depleting NTP pools, which would be fatal [76]. In fact, many of the changes discussed also impact each other, potentially synergizing to keep the cell in a safe, low-activity state: the conformation of supercoiled DNA is very sensitive to solute concentrations [40], topoisomerase and gyrase activity are affected by nucleoid associated proteins (NAPs, see below) [77,78], and gyrase activity is sensitive to ATP concentrations [43]. For any transcription at all to occur in this context, specific adaptations are likely to be needed to counter the changes to the energy landscape of transcription initiation and elongation imposed by growth arrest (Figure 1).

## Mechanisms to facilitate transcription during growth arrest

**Figure 2.**
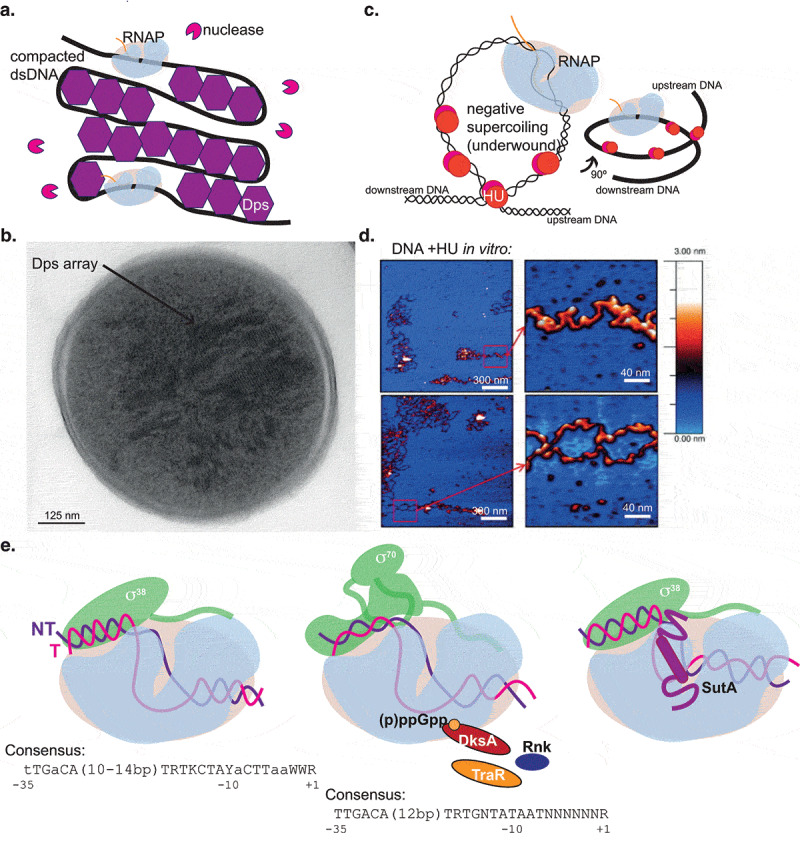
Factors modulating transcription during growth arrest. A. Nucleoid-associated proteins upregulated during growth arrest can modulate the nucleoid environment and DNA topology. Dps binds DNA in a crystalline-like lattice that excludes restriction enzymes but does not inhibit RNAP. B. Transmission electron microscopy of a stationary phase *E. coli* cell, showing Dps arrays. Reprinted with permission [89]; arrow indicating Dps and scale bar label added for clarity. C. The HU β subunit is upregulated in stationary phase, increasing the prevalence of the HU αβ heterodimer. HU can constrain negative supercoils, possibly affecting their distribution under a condition where average supercoil density is lower. D. Atomic force microscopy of purified DNA plus HU αβ heterodimer. Reprinted with permission [95]; scale bar labels have been added for clarity. E. RNAP-binding proteins can modulate RNAP behavior during growth arrest. Left: The stress sigma factor σ^38^ (compare to σ^70^, middle) is less sensitive to decreased supercoiling and increased solute concentrations than σ^70^ but can recognize similar promoter motifs (R = G/A; Y = C/T; W = A/T; K = G/T) [100]. Middle: Several factors can bind in the RNAP secondary channel, which allows access of NTPs to the active site. DksA/(p)ppGpp and TraR modulate the RNAP conformation, changing the energy landscape of initiation in a way that favors some promoters and disfavors others. Although Rnk seems able to bind at the same site, its effect on transcription is unknown. Right: SutA is present in Pseudomonadales species and binds the β1 domain of RNAP to affect transcription of many genes during growth arrest, slightly enhancing transcription of housekeeping genes including the rRNA operons

Many cells in prolonged growth arrest appear to spend extended periods of time engaging in little or no new transcription or protein synthesis (see single-cell transcriptome data below). However, low levels of new gene expression can be observed during prolonged starvation in multiple organisms [79] and may facilitate survival by allowing maintenance of essential cellular machinery, responses to threats, or incorporation of transiently available nutrients. Several global regulators have been identified as being specifically upregulated and/or functionally important in protracted growth arrest [80–83]. These factors can affect the nucleoid environment by binding to DNA with low sequence specificity (Figure 2a-d) or affect RNAP behavior by binding it directly (Figure 2e). Additionally, it seems likely that some core factors that are always present may alter their dynamics or interactions to adapt to growth arrest. A brief discussion of some of these factors can illustrate possible mechanisms by which the behavior of the transcription machinery might be altered in growth arrest, but many other such factors likely remain undiscovered – even in *E. coli*, over half of the genes in the genome do not have well-characterized functions, and genes with roles specific to protracted growth arrest are less likely to have been discovered in one of the growth-based screens that has dominated molecular microbiology.

The abundance and distribution of NAPs on the bacterial chromosome changes dramatically as cells enter growth arrest [84]. Here we focus on just two examples from protein families that are well-studied in *E. coli* but are also widely represented across the bacterial phylogeny. The roles of a range of NAPs have been previously reviewed [38,85]. Perhaps the most dramatic change is the induction of Dps (DNA-binding protein from starved cells), which was first identified in 1992 by ^35^S labeling of *E. coli* that had been starved for 3 days [86]. Dps becomes among the most abundant proteins in the cell in stationary phase and binds DNA in what appears by electron microscopy to be a strikingly regular crystalline lattice [87–89] (Figure 2a-b). It was initially proposed that an important consequence of this might be to hold the DNA in a transcriptionally inactive state that would be relatively protected from DNA damage during starvation [90]. While it’s true that Dps appears to contribute to condensing the nucleoid, which may in turn contribute to decreased average mobility and flexibility of DNA, a series of careful experiments recently revealed, surprisingly, that Dps does not inhibit transcription at all. Elongating RNAP is capable of transiently disrupting the associations between Dps and DNA, which are not sequence specific. Meanwhile, Dps does block access to DNA for restriction endonucleases, possibly fulfilling a protective role [91]. While the role of Dps during starvation remains incompletely understood, these observations suggest that preserving the DNA in a state that is accessible to the transcription machinery during growth arrest is a common strategy [92].

Members of the DNABII family of nucleoid-associated proteins may also play important roles during growth arrest. This family, which is considered functionally analogous to eukaryotic histones even though they lack sequence or structural homology, is widely conserved and many bacterial genomes encode multiple members. In *E. coli*, this includes IHF and HU, each encoded as alpha (*ihfA, hupA*) and beta (*ihfB, hupB*) monomers that can form homo- or heterodimers [93]. Their binding to DNA bends it dramatically and/or constrains negative supercoils [40,94,95] (Figure 2c-d). The cellular concentration of IHF increases at the entry to stationary phase [84], with widespread impacts on expression of metabolic and virulence-related genes that in the Gram-negative plant pathogen *Dickeya didantii* are mediated by modulating the distribution of supercoils throughout the chromosome [96]. While the overall concentration of HU does not change upon entry into stationary phase in *E. coli* [84], the ratio of the beta subunit relative to the alpha subunit increases dramatically, as does the prevalence of the heterodimer [97]. HupB has also been reported to be overrepresented among newly synthesized proteins in growth-arrested *Pseudomonas aeruginosa* [80]. As observed with Dps, HU does not appear to be able to block progression of an elongating RNAP because its affinity for DNA is too low [38], but it has substantial impacts on gene expression in multiple organisms [93,98]. Mutation of either HU monomer negatively impacts long-term starvation survival in *E. coli* even though neither single mutation causes exponential growth defects [97], and it may be that proteins that can manage the local positioning of negative supercoils are more critical under conditions of reduced average negative supercoiling throughout the genome.

The stress sigma factor σ^38^ clearly plays a critical role in directing expression of stress response genes during growth arrest, including upregulation of Dps [86]. A Tn-Seq screen in *P. aeruginosa* showed that it contributes significantly to fitness during growth arrest due to either oxygen or carbon deprivation [81]. Its functions in *E. coli* have been reviewed elsewhere [99,100], but a few points are worth making here. First, in multiple organisms σ^38^ does have a regulon of stress-specific genes whose promoters are not efficiently recognized by the σ^70^ housekeeping sigma factor, but there is substantial overlap in the consensus sequences recognized by σ^38^ and σ^70^ [100,101] (Figure 2e), and it is possible that a role of σ^38^ during protracted growth arrest could be low-level transcription of housekeeping genes as well. σ^38^ drives lower levels of transcription than σ^70^ from promoters that can be recognized by both [100], but *in vitro* and *in vivo* experiments have suggested that it is less sensitive to loss of negative supercoiling or increased solute concentrations [58,102]. In many organisms, σ^70^ holoenzyme is sequestered in stationary phase by binding to the 6S RNA, which mimics an open promoter complex, leaving σ^70^ holoenzyme perhaps mostly unavailable until a true nutrient upshift leads to its release from sequestered 6S complexes [103]. Second, while the intricate network of post-transcriptional regulators affecting σ^38^ expression has been extensively characterized in *E. coli*, the regulation appears to be substantially different even in other Gammaproteobacteria such as *P. aeruginosa* [104]. More careful explorations of transcription and translation within growth-arrested states of diverse organisms are now becoming feasible and should yield new insight.

No discussion of transcription regulatory factors important during growth arrest would be complete without mention of the stringent response small-molecule alarmone (p)ppGpp. (p)ppGpp is produced by nearly all bacteria in a variety of stressful circumstances, and affects replication, transcription, translation, and nucleotide biogenesis by diverse mechanisms [105–107]. In Gram-positive bacteria, exemplified by the model *B. subtilis*, (p)ppGpp does not bind RNAP. Instead, its effects on transcription appear to be mediated by its inhibition of GTP synthesis. Promoters downregulated by the stringent response (including all of the rRNA promoters that were directly investigated) have GTP as the initiating nucleotide, while upregulated genes have ATP as the initiating nucleotide. Inhibition of GTP synthesis can increase ATP levels, as both pathways draw from the same purine precursor pool [70,71,108]. In many Gram-negative bacteria on the other hand, (p)ppGpp binds directly to RNAP at two sites, one of which is formed by the binding of the global transcriptional regulator DksA in the secondary channel [109]. Binding of DksA and (p)ppGpp directly affects transcription of hundreds of genes, some positively and some negatively, leading to repression of ribosome biogenesis and upregulation of amino acid biosynthesis [110]. Interestingly, the conjugation-associated gene TraR also binds RNAP in the secondary channel, in a manner analogous to that of (p)ppGpp/DksA, and recent cryoEM structures of the TraR/RNAP holoenzyme complex on a ribosomal protein promoter have revealed how these binding events are likely to alter the conformation of the RNAP holoenzyme to shift the energy landscape of initiating transcription. Importantly, depending on the underlying energy landscape of the promoter in the absence of TraR (determined by DNA sequence, conformation, and interactions with additional regulators) the same TraR impacts on RNAP conformation can have positive or negative effects on initiation [15]. Many of the changes to the DNA and nucleoid that occur during growth arrest may also be viewed as changing the energy landscape of specific promoters in different ways, and growth arrest-specific regulatory factors must operate in this context.

Other less well-characterized RNAP-binding factors seem to increase in abundance during growth arrest or stationary phase and may also impact the conformation of RNAP and the energy landscape of its interactions with promoters in diverse ways. For example, a protein called Rnk, which bears structural similarity to the Gre transcription elongation factors and can compete with DksA for binding in the secondary channel [111], is upregulated during anoxia-induced growth arrest in *P. aeruginosa* [80]. Its impact on transcription in this condition is completely uncharacterized. Also in *P. aeruginosa*, a small, acidic, and largely unstructured protein named SutA was recently shown to be upregulated in stationary phase and anoxia [80]. SutA binds to the β1 domain (or protrusion) of RNAP, and modestly enhances transcription of hundreds of mostly housekeeping genes, including the rRNA genes [112]. This finding was initially counterintuitive, but if low levels of new protein synthesis are required during growth arrest to respond to threats or transient opportunities, factor(s) that modulate the energy landscapes of these promoters to counter the negative influences of growth arrest-induced changes to DNA conformation and nucleoid environment might be required.

In addition to questions about changes in specific regulators present during growth arrest, many questions remain about how changes to the dynamics of the whole system play out. While average transcriptional output is clearly greatly reduced during growth arrest, much less is known about how this is mediated at an individual cell or individual transcript level. Does elongation proceed at the same rate, but by many fewer RNAPs? Or do changes to the nucleoid density, DNA topology, or interactions with ribosomes (for example) cause elongation to proceed more slowly? To what extent can RNAP or ribosomes tolerate changes in elongation rates, and do transcription and translation remain coupled? Sequestration of ribosomes and RNAPs in inactive states does appear to be an important strategy in multiple organisms, especially in carbon starvation [103,113,114], and allowing activity of only a very small number of ribosomes and polymerases could in theory help support higher elongation rates by limiting competition for substrates. Studies in *E. coli* have suggested that changes to transcription and translation elongation rates versus numbers of active ribosomes differ depending on the nature of the starvation condition [114], and that transcription and translation elongation rates remain coordinated, at least for the model *lacZ* transcript [115].

The question of whether transcription and translation are physically coupled in diverse organisms and conditions remains of great interest. While structures from *E. coli* have shown direct physical contact between the RNAP and ribosome [116–118], recent work has suggested that transcription and translation are not tightly coupled in many Gram-positive bacteria, and that the transcription terminator Rho plays a less important role or is absent in these organisms [119]. Even in *E. coli*, there are structural, biochemical and genetic data to suggest that the RNAP and ribosome are not always physically coupled; a stable interaction may not even be the norm, but a trailing ribosome can catch up to and “push” a stalled RNAP [118,120,121]. New cryoEM structures have revealed in greater detail how the Rho hexamer can cause termination in the absence of a closely following ribosome by altering the conformation of RNAP [22]. Additional new structures have also shown how the ribosomal RNA anti-termination complex (composed of NusA, NusG, NusE, NusB, ribosomal protein S4, and SuhB) can protect the untranslated ribosomal RNA from this fate [122]. NusA and NusG have long been appreciated as transcription elongation factors that can modulate elongation on many transcripts besides the rRNA genes, but recent work has suggested that SuhB might also play more diverse roles as well. In enteric bacteria, it appears to regulate attenuation of its own transcript by Rho [123], and in *P. aeruginosa, suhB* deletion affects levels of several genes [124]. Interestingly, several members of the anti-termination complex are relatively more highly expressed during growth arrest compared to active growth in *P. aeruginosa* [80]. Another growing body of work has shown that interactions of sigma factors with elongating RNAP are more common than once thought and can modulate recruitment of elongation factors [125–127]. Exactly how these diverse elongation complexes may participate during growth arrest is unknown, but the recent structures will provide a foundation for evaluating hypotheses. Together, these observations suggest the possibility that the coordination between transcription and translation may be more flexible and adaptable than traditional models suggest.

## Single-cell transcriptome data and growth arrest

A major open question for understanding transcriptional regulation during growth arrest is how the very low levels of transcriptional activity are distributed over the cells of a bacterial population. Recent advances in measuring transcript abundances in single cells open exciting new opportunities for gaining insight into this question. Single-cell analysis of transcript abundance is much more challenging in bacteria than in eukaryotes due to very low transcript abundance (related to very small cell sizes and short mRNA half-lives), the lack of a polyA-tail by which to easily separate mRNA from rRNA, and the robust cell envelopes of bacteria. Since mid-2020, several different approaches have been used to look at single-cell transcriptomes of stationary phase cells (Table 1), and at least one additional method that not yet been used to look at stationary phase cells could be useful in the future [128].

**Table 1.** 

Strategy name	Description	Organisms examined in stationary phase	Ref
MATQ-Seq	single-cell sorting by FACS, followed by lysis and amplification of transcripts by “multiple annealing and dC-tailing-based quantitative scRNA-seq”	*S. enterica* Typhimurium	[133]
PETRI-Seq	split-pool barcoding of transcripts *in situ* within fixed cells	*E. coli*	[129]
microSPLiT	split-pool barcoding of transcripts *in situ* within fixed cells	*B. subtilis*	[130]
par-seqFISH	microscopy-based approach in which sequential fluorescent in-situ hybridizations with probes against mRNA transcripts allow direct counting of transcripts for 105 different genes	*P. aeruginosa*	[131]

These methods have different strengths and weaknesses; none of them capture complete information about the transcripts present in each bacterial cell investigated. For example, the sequencing-based approaches have no way of obtaining any information about the numbers of cells from which no transcripts were captured, potentially impacting analysis of the distribution of gene expression activity across a population where many cells are not active at all. Also, while these approaches will in theory make unbiased identifications of all transcripts present, capture efficiency is relatively low. Exponential phase *E. coli* cells, estimated to contain 10^3^–10^4^ transcripts, yielded a median of about 230 transcripts by the two split-pool barcoding methods [129,130], for example. In contrast, the par-seqFISH method can only detect transcripts from the genes pre-selected for probe design and production, and the requirement to design multiple non-overlapping probes per gene limits its ability to detect very short transcripts. However, fluorescence *in situ* hybridization (FISH)-based methods are the current gold standard for detection of individual transcripts, allowing direct counting of transcripts per cell for the genes probed, including identification of cells containing none of the probed transcripts. Furthermore, additional morphological properties of cells can be associated with their transcriptome profiles [131].

A first observation from stationary phase cells in each of these experiments is that they do not contain large numbers of transcripts. The split-pool methods detected a median of 27 and approx. 25–30 transcripts per cell for *E. coli* and *B. subtilis*, respectively [129,130]. The par-seqFISH method identified a median of 9 transcripts per cell (probing for only 105 genes) [131]. Assuming that transcript capture efficiency is similar for stationary phase and exponential phase in the split-pool methods, and that the 105 probed transcripts are representative of all transcripts in the genome for the par-seqFISH method, these measurements predict actual median transcript numbers of just 300–500 per cell. Of course, some cells in a stationary phase population have many more transcripts; the most transcript-rich cells in the par-seqFISH experiment had 16-fold more transcripts than the median [131].

Some common themes emerge in the identities of transcripts expressed by stationary phase cells. In all datasets, the most abundant transcripts included proteases, peptidases, and transporters with putative functions in scavenging diverse nutrients from the environment. Genes involved in amino acid biosynthesis and degradation pathways were also expressed. All four data sets also showed some expression of nitrate and/or nitrite reductase genes, potentially important at high cell densities as oxygen becomes limiting [132]. Interestingly, the *S. enterica* Typhimurium, *B. subtilis*, and *P. aeruginosa* datasets also show very high relative expression of some motility-related genes (for surfactin production in *B. subtilis*, and flagella in *P. aeruginosa* and *S*. Typhimurium), consistent with a model that some cells might search for a new niche in response to starvation [130,131,133]. *P. aeruginosa* and *S*. Typhimurium both produce Type III secretion system effectors in stationary phase [131,133]. Finally, while σ^38^ (*rpoS*) transcripts were detected in both *E. coli* and *P. aeruginosa*, they were by far the most abundant transcripts among all genes assayed in the *P. aeruginosa* dataset, in three different stationary phase-like conditions. This may be an example of a gene whose transcript levels are disconnected from its protein levels in stationary phase. In one proteomics study of growth arrested *P. aeruginosa*, new RpoS protein synthesis was not detected [80], and in a second study where proteomes of cells highly expressing an *rpoS* transcriptional reporter were selectively investigated, RpoS protein levels were not particularly high [82]. Further investigation of this regulation is needed.

**Figure 3.**
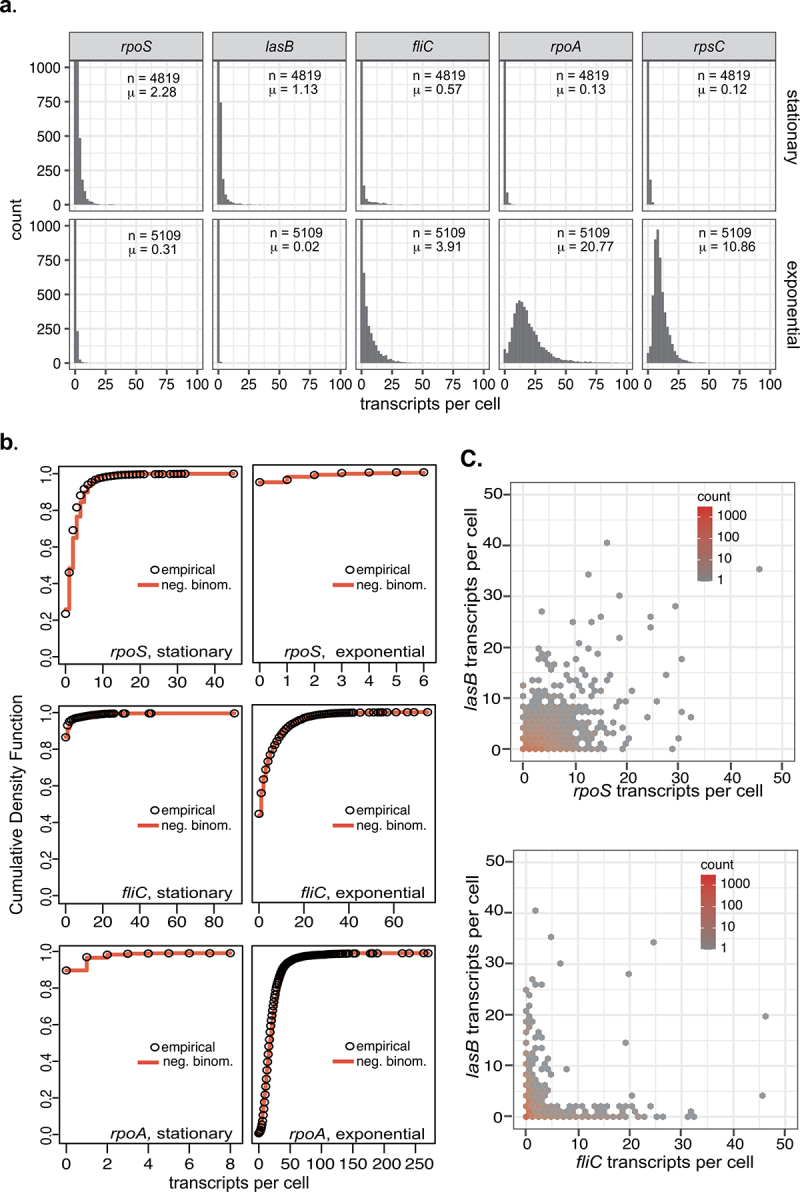
Distributions of transcripts per cell in stationary and exponential phase *P. aeruginosa*. A. Histograms showing distributions of transcripts per cell for five different genes in stationary and exponential phase. Numbers of cells (n) and arithmetic means of expression levels (μ) are indicated. All cells were from the LB dataset. Stationary phase used the OD = 3.2 timepoint, and exponential phase combined the OD = 0.2 and OD = 0.45 timepoints, which are both traditionally considered exponential phase, to achieve a similar total number of cells. B. Plots comparing empirical cumulative density functions to theoretical cumulative density functions from the negative binomial distribution fitted to the data. Distributions were fitted using the fitdistrplus package in R. C. Comparison of numbers of two different transcripts in each cell. Each hexbin covers approximately one possible count value; the number of cells falling into that bin is represented by color. Two cells fall outside the boundaries of these plots. Even though these were among the most abundant transcripts in stationary phase, most cells still had 0 copies of each of them. All plots were generated using the ggplot2 package in R, using data from Dar *et al*. [131]

A unique opportunity with a single-cell data set is to evaluate how transcripts for expressed genes are distributed across the population of single cells. Because the par-seqFISH analysis does not discard cells with few transcripts, it is perhaps best suited to further exploration of the distributions of per-cell transcript numbers for specific genes in stationary phase. Using the publicly available par-seqFISH dataset from stationary phase in LB [131], histograms of per-cell transcript counts were calculated for three of the most highly expressed genes in stationary phase (*rpoS, lasB*, and *fliC*) and two of the most highly expressed genes in exponential phase (*rpoA* and *rpsC*) (Figure 3a). Even for the most highly expressed genes in stationary phase, the most common transcript count per cell was 0. In contrast, a large majority of cells in exponential phase contained at least one transcript each for *rpoA* and *rpsC*. Interestingly, some stationary phase cells also contained transcripts for these genes (encoding RNAP and ribosome subunits), and in fact they were still among the top quartile of expression levels in stationary phase. Even though the shapes of the histograms are very different, the transcript count data is well fitted by a negative binomial distribution in each case (Figure 3b). The negative binomial distribution has previously been fitted to transcript numbers in single-cell sequencing experiments, and modeling to link this observation to an underlying mechanistic basis suggested that bursts of transcription could yield such a pattern [134]. Many bacterial promoters have previously been shown to have “bursty” dynamics during exponential growth [135], and in stationary phase, such dynamics may be even more pronounced, especially if the difficulties of managing DNA supercoiling are exacerbated [136].

Finally, in general, the correlation between the numbers of any two tested transcripts was relatively low. This is perhaps to be expected if transcript numbers per cell are very low and mRNA half-lives remain short – cells may not be able make transcripts for all the genes in a large regulon at the same time (Figure 3c). Several studies have suggested that mRNA half-lives for at least some transcripts may increase under conditions of slow growth, energy limitation, and growth arrest in diverse organisms, but many questions remain about how this contributes to gene expression dynamics [75,137,138]. *rpoS* and *lasB* (elastase, a secreted protease), two of the most highly expressed genes, were among the more highly correlated, and *rpoS* has previously been shown to contribute to *lasB* regulation [101,139]. On the other hand, *fliC* (flagellin, a structural component of flagella [140]), another relatively highly expressed transcript, was rarely present in the same cell as *lasB*. Understanding the regulatory mechanisms allowing gene expression to be distributed over different cells of a population, and also over time, to optimize fitness under extreme limitation, will be a compelling challenge for future work.

The ability to measure specific transcript numbers in single cells is a powerful tool to further probe the low and heterogeneous transcriptional activity that characterizes growth arrest in bacteria. The existing rich structural and biochemical understanding of the core transcriptional machinery is another important resource that is foundational for further exploration of growth arrest. While there are many good reasons why most efforts to study the transcriptional machinery have focused on rapid exponential growth or the initial responses to a disruption, our expanding recognition of the presence of bacteria in every environment forces us to acknowledge that many of them spend most of their time not actively dividing. Furthermore, starved and growth-arrested bacteria are likely to carry out many of their important functions for pathogenesis, symbiosis, or their own survival, while they are still growth arrested. Thus, a better understanding of how transcription operates during growth arrest will be key for making progress toward sustainable agriculture, infection control, and solving the many other pressing problems in which the ubiquitous bacteria of our planet play a part.
